# Takotsubo Cardiomyopathy and QTc Prolongation with Subsequent Improvement of QTc Interval and Resolution of Apical Ballooning: A Case Report

**DOI:** 10.7759/cureus.9143

**Published:** 2020-07-11

**Authors:** Violeta Alvarez Retamales, Odalys E Lara Garcia, Suhayb Ranjha, Cameron Koester, Mohamed R Labedi

**Affiliations:** 1 Internal Medicine, Southern Illinois University School of Medicine, Springfield, USA; 2 Internal Medicine: Cardiology, Southern Illinois University School of Medicine, Springfield, USA

**Keywords:** stress-related cardiomyopathy, prolonged qtc interval, torsades de pointes, takotsubo cardiomyopathy

## Abstract

Stress-induced cardiomyopathy, also known as Takotsubo cardiomyopathy (TTS), is characterized by transient regional systolic dysfunction. Furthermore, electrocardiogram (ECG) changes can vary as TTS evolves. We report a case of a 67-year-old woman who presented to the ER after cardiac arrest. She was found to have stress-induced cardiomyopathy with prolonged QTc interval. The patient developed torsades de pointes for which she required cardioversion, followed by improvement of QTc interval corresponding to resolution of echocardiographic evidence of apical ballooning.

## Introduction

Takotsubo cardiomyopathy (TTS) is characterized by sudden left ventricular dysfunction and wall motion abnormalities involving the apical and mid segments [1]. TTS has been previously associated with prolongation of QT interval and increased risk of torsades de pointes, but resolution of QT interval prolongation with improvement of the echocardiogram findings is yet to be widely published [2]. Furthermore, usually with the resolution of the apical ballooning, there is an increase in the incidence of arrhythmia likely related to remodeling, yet on this case the opposite actually occurred. We report a case of stress-induced cardiomyopathy with prolonged QTc interval, and subsequent improvement of QTc interval corresponding to resolution of echocardiographic evidence of apical ballooning.

## Case presentation

A 67-year-old woman, with a history of coronary artery disease (CAD) status post coronary artery bypass graft (CABG), alcoholic cirrhosis, and depression presented to the ED after cardiac arrest. She arrived by ambulance after being found unconscious and required two cycles of cardiopulmonary resuscitation (CPR) in conjunction with two defibrillations for ventricular fibrillation along with epinephrine 1 mg given once, and then one cycle of CPR for pulseless electrical activity cardiac arrest achieving return of spontaneous circulation with no other medications administered. The patient was intubated for airway protection upon arrival to the ER and she was started on norepinephrine vasopressor therapy. Off note, her medication list included: aspirin, metoprolol tartrate, pravastatin, furosemide, metolazone, spironolactone, potassium chloride supplement, and citalopram.

**Figure 1:**
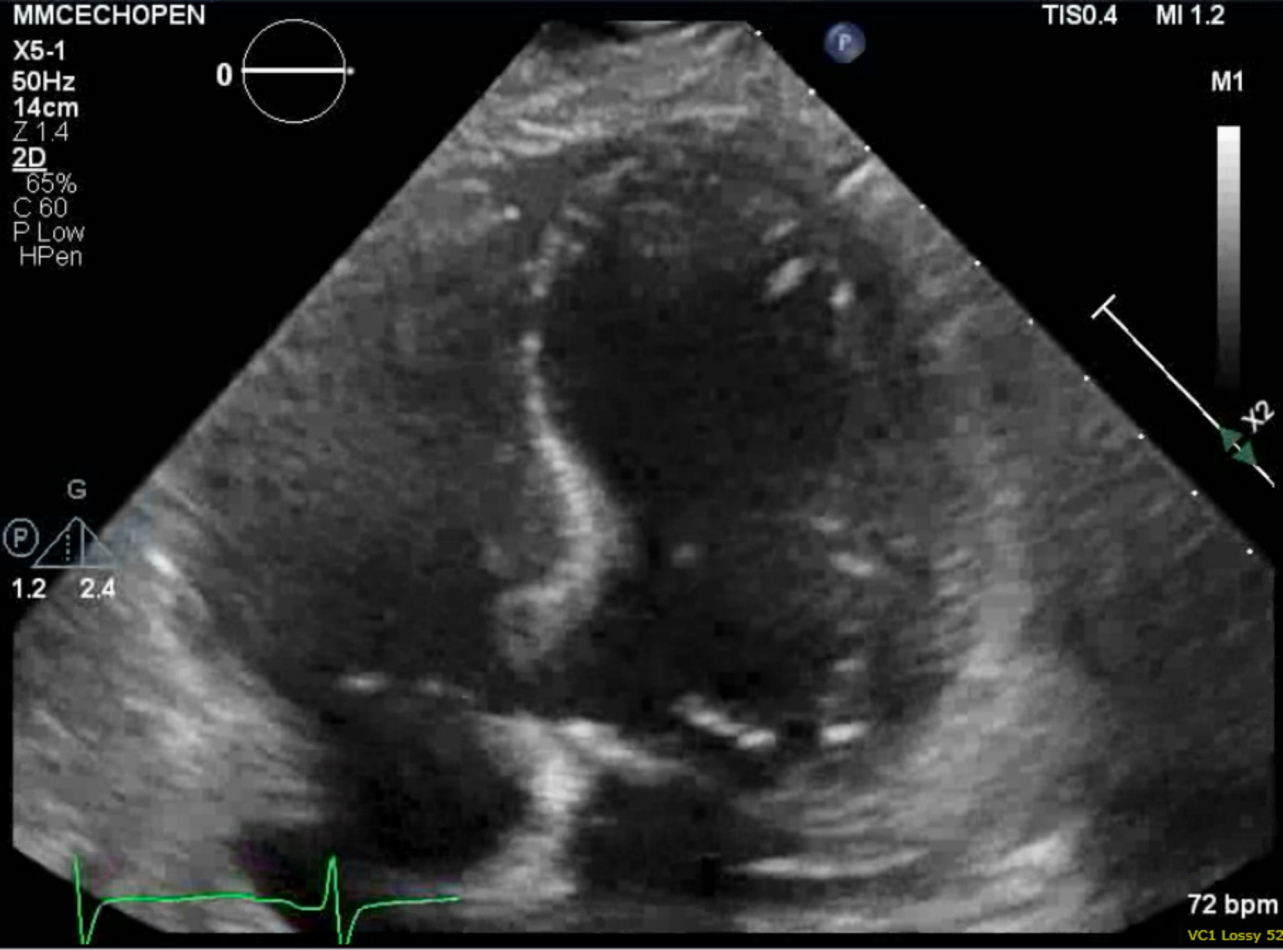
Transthoracic echocardiogram apical four chamber view showing left ventricular apical ballooning during systole.

**Figure 2:**
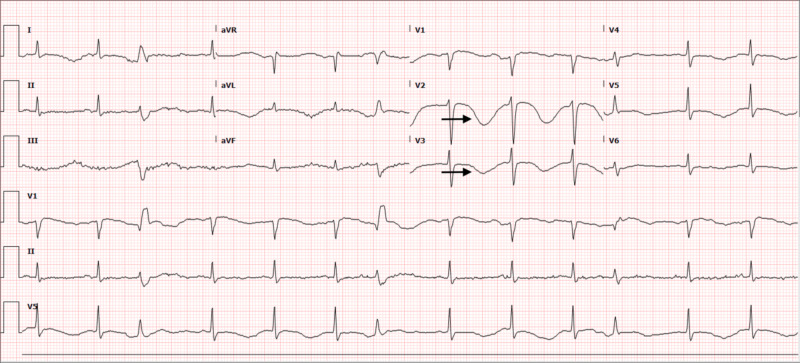
Admission ECG: QTc at 757 ms, deeply inverted T-waves on V2 and V3, and quadrigeminy arrhythmia. ECG, electrocardiogram

Laboratory testing was significant for hypokalemia of 2.2 mmol/L, other electrolytes were within normal limits. Transthoracic echocardiogram showed left ventricular apical dyskinesis and left ventricular ejection fraction (LVEF) of 30%-35% (Figure 1). ECG showed profound QTc prolongation above 700 ms and deeply inverted T-waves in the antero-lateral leads (Figure 2). The patient underwent postcardiac arrest workup, including CT angiography of the chest, with unremarkable results. Therefore, her cardiac arrest was thought to be secondary to her electrolyte imbalance. A day after her admission she became hemodynamically unstable as she went into monomorphic ventricular tachycardia with intermittent episodes of torsades de pointes for which she received synchronized cardioversion and was started on IV lidocaine. The patient’s potassium level one day after her admission remained decreased at 2.6 mmol/L despite aggressive repletion for which she was started on continuous IV potassium. In an attempt to suppress adrenergic stimulation, neuromuscular paralysis was initiated with cisatracurium. During the next 48 hours, the patient’s ventricular tachycardia converted to atrial fibrillation with a rapid ventricular response followed by normal sinus rhythm. The patient was able to be extubated to nasal cannula. Coronary angiography showed nonobstructive CAD with patency of left internal mammary artery to left anterior descending artery and Saphenous vein graft to first obtuse marginal branch along with patent stent in the proximal right coronary artery.

**Figure 3:**
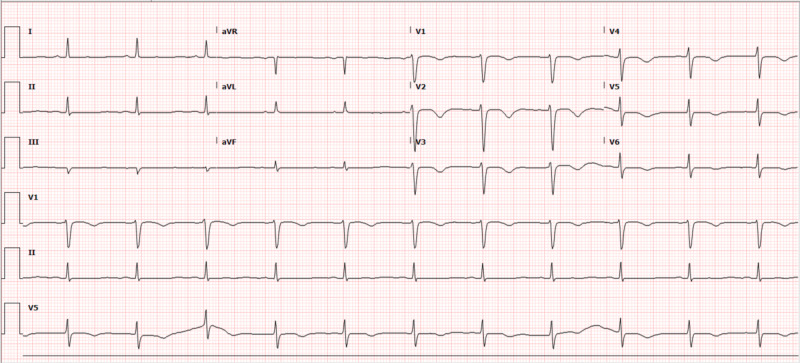
ECG five days after admission. QTc improved to 464 ms. T-wave inversion present in precordial leads. ECG, electrocardiogram

**Figure 4:**
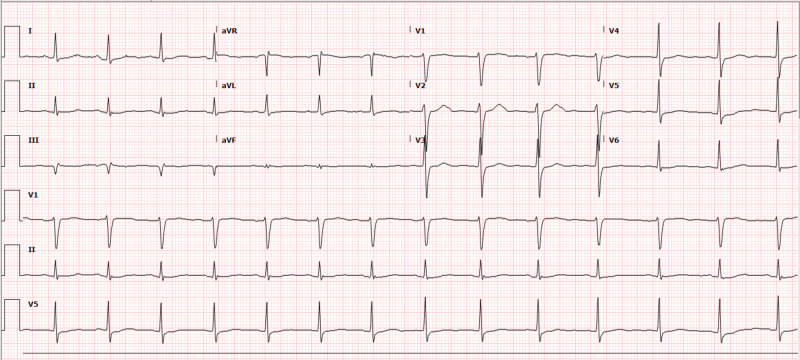
ECG six months after admission showing resolution of T-wave inversion on precordial leads and QTc at 369 ms. ECG, electrocardiogram

Serial ECGs showed improvement of QTc interval to 464 ms with persistent T-wave inversion with associated resolution of apical dyskinesis and normalization LVEF on echocardiogram by day 5 along with resolution of hypokalemia (Figure 3). The patient was discharged to an extended care facility after 19 days of hospitalization as her stay was complicated by pseudomembranous colitis. The patient received secondary prevention implantable cardioverter-defibrillator (ICD) as a means to prevent recurrent sudden cardiac arrest. The ECG done six months afterwards revealed resolution of prolonged QTc at 369 ms and resolved T-wave inversions (Figure 4).

 

## Discussion

Takotsubo cardiomyopathy is characterized by a sudden left ventricular dysfunction and wall motion abnormalities. In most cases of TTS, the regional wall motion abnormality extends beyond the territory perfused by a single epicardial coronary artery. Despite mimicking ST-segment elevation myocardial infarction (MI), TTS pathophysiology has not yet been fully elucidated. Postmenopausal women are more frequently affected than the other subpopulations as in this case [3]. Furthermore, there are studies which describe the ECG changes in TTS but knowledge regarding its prognostic significance is yet to be attained [2]. In this regard, various possible mechanisms -- LV outflow tract obstruction, including catecholamine-mediated cardiac toxicity, aborted MI caused by transient left anterior descending artery (LAD) occlusion, multivessel coronary spasm, and microvascular dysfunction -- have been postulated.

The ECG patterns of patients with TTS are diverse, and the prevalence of QTc prolongation is quite variable, ranging from 26% to 51% [4]. The International Takotsubo Registry study has demonstrated that the prevalence of QTc prolongation on the admission ECG is higher in patients with TTS compared to acute coronary syndrome [2]. The QTc prolongation in TTS is considered to reflect the transient myocardial insult and occurs secondary to a reduced repolarization reserve and intramyocyte calcium overload [4]. Therefore, severe repolarization abnormalities in TTS typically do not occur until 24-48 hours after presentation [4]. However, in this case, severe QTc prolongation was present on admission. This may be explained in part by hypokalemia, which can increase the risk of QTc prolongation, yet it can be a direct effect of TTS. Our patient not only had QTc prolongation but also ventricular arrhythmias which are rare, occurring in 4%-9% of cases [5]. The QTc prolongation resolution was seen six months along with persistence of TTE findings, example that ECG changes might persist for weeks [6].

The Taskforce on Takotsubo Syndrome of the Heart Failure Association recently proposed new criteria for TTS, including new and reversible ECG abnormalities during the acute phase [1]. This could aid many physicians as it is a major challenge when a patient presents with chest pain and an elevated ST segment on ECG, especially in older women where TTS is more prevalent but an acute coronary syndrome should be ruled out [7]. We were able to find a handful of cases where QTc prolongation was present, yet, in this case, serial ECGs showed progressive improvement of QTc interval with simultaneous resolution of echocardiographic findings within a couple of days illustrating the “transient” nature, classically seen on TTS.

## Conclusions

We were able to appreciate the transient nature of stress-induced cardiomyopathy with improvement of QTc interval on serial ECG with simultaneous resolution of apical hypokinesis on echocardiogram. The temporal association between TTS and QTc prolongation has yet to be widely studied and further investigations are warranted as it could result in changes in its management, leading to an improvement of morbidity and mortality rates.
